# Efficacy of aromatherapy for reducing stress among elderly residents in geriatric homes at Gujarat

**DOI:** 10.6026/973206300213791

**Published:** 2025-10-31

**Authors:** Navaneetha Krishnan A, Siva Subramanian N, Mahalakshmi B

**Affiliations:** 1 Department of Psychiatric Nursing, Nootan College of Nursing, Sankalchand Patel University, Visnagar, Gujarat, India; 2 Department of Paediatric Nursing, Nootan College of Nursing, Sankalchand Patel University, Visnagar, Gujarat, India

**Keywords:** Aromatherapy, lavender, stress, elderly, geriatric homes

## Abstract

Elderly residents in geriatric homes frequently experience stress and aromatherapy is a safe non-pharmacological intervention. A pre-experimental one-group pre-test post-test study was conducted among 50 elderly residents in Gujarat, who received daily lavender oil inhalation for 14 days. Stress was assessed using the Perceived Stress Scale and mean stress scores dropped significantly from 2.74 to 1.10 (p < 0.001). Duration of stay and nature of admission were significantly associated with pre-test stress. Thus, we show that lavender aromatherapy is effective, safe and affordable for reducing stress in institutionalized elderly populations.

## Background:

Stress is a prevalent psychological concern among elderly individuals, particularly those living in institutional settings such as geriatric homes. Aging often brings stress-inducing factors such as declining physical health, cognitive impairment, social isolation, bereavement and loss of autonomy [1]. These challenges significantly reduce quality of life and exacerbate medical conditions. A study in Punjab reported that the majority of elderly residents in old age homes experienced moderate stress levels, highlighting their psychological vulnerability [2]. Pharmacological interventions are often used to manage stress but may cause complications in older adults due to polypharmacy and side effects. Hence, there is increasing emphasis on safer, non-pharmacological alternatives such as aromatherapy, which are holistic, cost-effective and accessible [3]. Aromatherapy involves the inhalation or application of essential oils to stimulate olfactory receptors and influence the limbic system, thereby promoting relaxation and reducing anxiety [4]. Evidence from long-term care hospitals showed that aromatherapy massage with lavender oil significantly reduced salivary stress markers among elderly women [5]. A meta-analysis of 21 studies confirmed that aromatherapy has a moderate-to-large effect in lowering psychological stress, especially when combined with massage [6]. Similarly, a randomized controlled trial demonstrated significant stress reduction through lavender inhalation therapy in older women [7]. Community-based studies also associated aromatherapy with improved mental health and reduced stress among seniors [8] and it proved beneficial in stress management during the COVID-19 crisis in geriatric wards [9]. Therefore, it is of interest to report the efficacy of aromatherapy in reducing stress among elderly residents in geriatric homes.

## Methodology:

## Research design:

This study employed a Pri-experimental one group pre-test and post-test design to assess the effectiveness of aromatherapy in reducing stress among elderly individuals living in geriatric homes. A single experimental group was assessed before and after the intervention without the inclusion of a control group, making it suitable for naturalistic care settings.

## Setting of the study:

The research was conducted in two selected geriatric homes in Gujarat, India. These institutions were chosen based on administrative approval, availability of elderly residents meeting inclusion criteria and the feasibility of conducting supervised therapy sessions in a calm and controlled environment.

## Sample and sampling technique:

A total of 50 elderly participants aged 60 years and above were selected using a purposive sampling technique. Inclusion criteria required participants to be permanent residents of the geriatric home for at least six months, capable of giving informed consent and experiencing mild to moderate stress levels as determined through baseline screening. Participants with severe cognitive impairment, psychiatric illness, hypersensitivity to essential oils, or those undergoing concurrent psychological treatments were excluded.

## Description of the intervention:

The intervention involved aromatherapy using lavender essential oil, administered through inhalation. Participants were exposed to 2-3 drops of lavender oil placed on a cotton pad positioned near their pillow or under the nose for 20 minutes daily over a 14-day period. All sessions were conducted in a quiet, supervised environment within the geriatric home to ensure comfort and safety.

## Data collection tool:

Stress levels were measured using the Perceived Stress Scale (PSS), a validated 10-item instrument widely used to assess perceived stress in adults, including the elderly. The scale has demonstrated high reliability and constructs validity in previous geriatric-focused research.

## Ethical considerations:

Ethical approval was obtained from the Institutional Ethics Committee. Written informed consent was secured from each participant after explaining the purpose, procedure and voluntary nature of the study. Participants were assured of their right to withdraw at any time and all data were anonymized to protect confidentiality.

## Statistical analysis:

Data analysis was carried out using SPSS software (version 26). Descriptive statistics such as frequency, percentage, mean and standard deviation was used to describe the demographic characteristics and stress scores. A paired t-test was used to compare the pre-and post-test stress scores, with p-values < 0.05 considered statistically significant.

## Results:

Tables 1 (see PDF) and 2 (see PDF) present the key findings on the effectiveness of aromatherapy in reducing stress among elderly residents in geriatric homes. Table 1 (see PDF) shows a statistically significant reduction in stress levels following a 14-day aromatherapy intervention using lavender oil, with the mean stress score dropping from 2.74 (pre-test) to 1.10 (post-test) and a highly significant p-value of 0.000. This indicates that aromatherapy had a strong positive impact on lowering stress among participants. Table 2 (see PDF) further explores the association between pre-intervention stress levels and demographic variables. While most variables, such as age, gender and education, did not show significant associations, two factors-duration of stay in the geriatric home (p = 0.008) and nature of admission (voluntary vs. forced, p = 0.021)-were significantly related to stress levels. These findings suggest that institutional factors, such as how and how long a person has lived in the facility, may influence their psychological stress and aromatherapy can serve as an effective intervention regardless of these variables. Figure 1 (see PDF) shows that before aromatherapy, 37 participants reported high stress and 13 had moderate stress, whereas after aromatherapy, 45 participants reported low stress and only 5 remained in the moderate category.

## Discussion:

This study aimed to evaluate the effectiveness of aromatherapy in reducing stress among elderly residents in geriatric homes in Gujarat. The findings align with a growing body of evidence that supports the use of aromatherapy as a non-pharmacological intervention for stress management in older adults. Several studies affirm that aromatherapy, particularly when used with massage or inhalation techniques, can significantly reduce psychological stress in the elderly. For example, a quasi-experimental study in Japan demonstrated that lavender aromatherapy massage effectively reduced salivary amylase activity, a biological marker of stress, among elderly women in long-term care facilities [10]. This supports the current study's findings, where a similar lavender oil intervention led to significant reductions in Perceived Stress Scale (PSS) scores. Aromatherapy's ability to stimulate olfactory receptors and influence the limbic system provides a plausible physiological basis for its stress-reducing effects. In a 2022 study by Ke and colleagues, compound essential oil inhalation significantly improved both physical and mental health among middle-aged and elderly community members in Taiwan, including measurable reductions in stress [8]. Additionally, a randomized study by Tang and Tse (2014) in community-dwelling older adults confirmed aromatherapy's ability to reduce depression, anxiety and stress, thereby enhancing emotional well-being [11]. Interestingly, novel interventions combining aromatherapy with digital technology have emerged. Cheng *et al.* (2020) combined hands-on aromatherapy with 3D virtual reality and reported significant reductions in perceived stress and improvements in happiness and sleep quality among elderly participants [12]. Some studies also assessed aromatherapy's effects in frail or cognitively impaired elders. A Taiwanese trial showed that lavender aromatherapy reduced loneliness and depression in frail elderly participants attending day-care centers [13]. Another study focusing on the Japanese herb Lindera lancea showed mood stabilization and improved relaxation in long-term care clients [14]. These inconsistencies highlight the importance of controlled intervention design and standardization of aromatherapy protocols. Additionally, the mode of delivery (inhalation vs. massage), type of essential oil, frequency and duration of intervention influence outcomes. For example, Her and Cho (2021) noted that aroma massage sessions lasting more than 20 minutes, conducted over ≤4 weeks, yielded the largest improvements in sleep and stress reduction [15]. The psychological effects of aromatherapy have also been shown to translate into improvements in sleep, a common challenge among the elderly. A study by Joseph and Joseph (2016) found that lavender aromatherapy significantly improved sleep quality in elderly individuals living in old age homes, as measured by the Pittsburgh Sleep Quality Index (PSQI) [16]. These results are promising given the relationship between poor sleep and heightened stress. The current study's findings are further supported by numerous investigations highlighting the benefits of lavender aromatherapy among the elderly [17]. Sigaroudi *et al.* (2021) found consistent improvements in sleep quality in older adults across six studies using various lavender delivery methods [17]. Lavender's mental health effects are also notable. Can *et al.* (2024) found significant reductions in anxiety and improvements in cognitive function and sleep among elderly diabetics using lavender and rosemary oils [18]. Yildrilim *et al.* (2025) confirmed lavender's effectiveness in just seven nights of use, while Apriyanti *et al.* (2025) observed a dramatic drop in insomnia scores (p = 0.001) after intervention [19-20]. Additionally, Xu *et al.* (2024) conducted a systematic meta-analysis showing that lavender-based non-inhalation aromatherapy delivered for less than four weeks produced a strong positive effect on sleep quality (SMD = -1.39) and also significantly reduced anxiety and depression in older adults [21]. Finally, it is essential to address that aromatherapy remains a complementary intervention. While effective, it should not replace comprehensive psychological or psychiatric care when required. Nevertheless, the consistently positive outcomes across diverse settings suggest aromatherapy holds strong potential as a supportive measure in geriatric mental health care.

## Conclusion:

Aromatherapy is a valuable, non-invasive and cost-effective strategy to reduce stress among elderly populations, particularly in institutional settings. Future research should focus on optimizing delivery methods, exploring cultural preferences in essential oils and incorporating longer follow-up to assess sustained benefits.
